# NKX6-1 mediates cancer stem-like properties and regulates sonic hedgehog signaling in leiomyosarcoma

**DOI:** 10.1186/s12929-021-00726-6

**Published:** 2021-04-27

**Authors:** Po-Hsuan Su, Rui-Lan Huang, Hung-Cheng Lai, Lin-Yu Chen, Yu-Chun Weng, Chih-Chien Wang, Chia-Chun Wu

**Affiliations:** 1 Translational Epigenetics Center, Shuang Ho Hospital, Taipei Medical University, New Taipei City, Taiwan; 2 Department of Obstetrics and Gynecology, Shuang Ho Hospital, Taipei Medical University, New Taipei City, Taiwan; 3 Department of Obstetrics and Gynecology, School of Medicine, College of Medicine, Taipei Medical University, Taipei, Taiwan; 4 Department of Obstetrics and Gynecology, Tri-Service General Hospital, National Defense Medical Center, Taipei, Taiwan; 5 Department of Orthopaedics, Tri-Service General Hospital, National Defense Medical Center, Neihu District, No. 325, Sec. 2, Chengong Road, Taipei, 11490 Taiwan

**Keywords:** Leiomyosarcoma (LMS), NK6 homeobox 1 (NKX6-1), Sonic hedgehog (SHH) signaling, SHH inhibitor, Chemoresistance

## Abstract

**Background:**

Leiomyosarcoma (LMS), the most common soft tissue sarcoma, exhibits heterogeneous and complex genetic karyotypes with severe chromosomal instability and rearrangement and poor prognosis.

**Methods:**

Clinical variables associated with NKX6-1 were obtained from The Cancer Genome Atlas (TCGA). NKX6-1 mRNA expression was examined in 49 human uterine tissues. The in vitro effects of NXK6-1 in LMS cells were determined by reverse transcriptase PCR, western blotting, colony formation, spheroid formation, and cell viability assays. In vivo tumor growth was evaluated in nude mice.

**Results:**

Using The Cancer Genome Atlas (TCGA) and human uterine tissue datasets, we observed that *NKX6-1* expression was associated with poor prognosis and malignant potential in LMS. *NKX6-1* enhanced in vitro tumor cell aggressiveness via upregulation of cell proliferation and anchorage-independent growth and promoted in vivo tumor growth. Moreover, overexpression and knockdown of *NKX6-1* were associated with upregulation and downregulation, respectively, of stem cell transcription factors, including *KLF8*, *MYC*, and *CD49F*, and affected sphere formation, chemoresistance, NOTCH signaling and Sonic hedgehog (SHH) pathways in human sarcoma cells. Importantly, treatment with an SHH inhibitor (RU-SKI 43) but not a NOTCH inhibitor (DAPT) reduced cell survival in *NKX6-1*-expressing cancer cells, indicating that an SHH inhibitor could be useful in treating LMS. Finally, using the TCGA dataset, we demonstrated that LMS patients with high expression of *NKX6-1* and *HHAT,* an SHH pathway acyltransferase, had poorer survival outcomes compared to those without.

**Conclusions:**

Our findings indicate that *NKX6-1* and *HHAT* play critical roles in the pathogenesis of LMS and could be promising diagnostic and therapeutic targets for LMS patients.

**Supplementary Information:**

The online version contains supplementary material available at 10.1186/s12929-021-00726-6.

## Background

Leiomyosarcoma (LMS) is a malignant mesenchymal neoplasm of the smooth muscle lining, occurring most frequently in the uterus, retroperitoneum, extremities, and other primary sites throughout the body [1]. LMS accounts for approximately 24% of soft tissue sarcomas [2] and presents largely as high-grade tumors, which exhibit aggressive behavior and a high propensity for local recurrence and metastasis via hematogenous spread [3]. Uterine LMS accounts for approximately 60% of all uterine sarcomas [4]. LMS is conventionally treated with surgical resection, adjuvant chemotherapy, and radiotherapy [2, 5]. However, systemic treatment for uterine LMS demonstrates only a modest response with approximately 40% metastasis [4, 6] and a 5 year survival rate of approximately 50% in patients with uterus-confined tumors [7]. The median overall survival is less than two years with doxorubicin- or gemcitabine-based therapies [8, 9]. Adjuvant pelvic radiation is not associated with significant improvement in the local control rate [10]. Even with combination regimens, treatment typically fails due to local recurrence and metastasis, resulting in a dismal prognosis. [7]. Such evidence indicates the malignant behavior of LMS and the urgent need for novel treatment options. In addition, the molecular mechanisms of LMS etiology are heterogeneous and are associated with diverse cytogenetic lesions. Consequently, elucidation of LMS pathogenesis is critical, especially considering that no epithelial precursor lesion of origin is known. Since the benefits of chemotherapy and radiotherapy for recurrent and metastatic disease are limited, an in-depth understanding of molecular disease mechanisms may provide a key to new therapeutics.

The NKX family of homeodomain-containing transcription factors is heavily involved in tissue-specific differentiation and development. Accumulating evidence indicates that these transcription factors are associated with a variety of cancers, including sarcomas. More specifically, *NKX6-3* depletion results in multiple genetic mutations that drive carcinogenesis of the stomach [11], while NKX2-5 regulates thyroid cell differentiation in thyroid cancer [12]. NKX2-1 mutations have been found in 16% of lung cancers [13] and are associated with lung cancer metastasis [14, 15]. NKX3-1 deletion and promoter hypomethylation have been identified in prostate cancer [16]. The NKX6-1 gene product interacts directly with the oncogenic EWS/ATF-1 chimeric transcription factor in clear cell sarcoma [17]. Moreover, hypermethylation of the NKX6-1 promoter is frequently detected in leukemia, cervical cancer, ovarian cancer and colon cancer [18–21]. Additionally, NKX2-2 was identified as a target of EWS-FLI-1, the fusion protein that is now considered a biomarker for Ewing sarcoma [22]. These results highlighted the critical role of the NKX family in the pathogenesis of cancer development and prompted us to investigate the previously undefined role of the NKX family in the initiation and progression of LMS.

In the present study, we investigated the potential role of the NKX family in LMS using human sarcoma cell lines, The Cancer Genome Atlas datasets, and uterine tissues from 49 patients. Our results demonstrated that NKX6-1 acts through the sonic hedgehog (SHH) pathway to increase cell proliferation, drug resistance, and cancer stemness in vitro and tumorigenicity in vivo. Importantly, inhibition of the SHH pathway significantly inhibits the growth of sarcoma cells with high NKX6-1 expression, indicating possible new treatment options for LMS patients.

## Methods

### Clinical samples

Tissue samples were collected with the informed consent of patients at the Tri‐Service General Hospital, National Defense Medical Center, Taipei, Taiwan. The samples included 19 LMS, 14 nonmalignant leiomyomas and 16 normal myometrium. These specimens were obtained during surgery, frozen immediately in liquid nitrogen and stored at − 80 °C until analysis. This study was conducted in accordance with the Declaration of Helsinki and approved by the Institutional Review Board of the Tri-Service General Hospital. All of the patients signed informed consent forms before the study.

### Cell lines

Two human uterine sarcoma cell lines (MES-SA and MES-SA/DX5) were obtained from BCRC (Bioresource Collection and Research Center, Taiwan). Cells were cultured at 37 °C and 5% CO_2_ in McCoy's 5a medium (Invitrogen) supplemented with 10% fetal bovine serum (Biological), 100 U/mL penicillin, and 100 μg/mL streptomycin.

### Transfections and NKX6-1 overexpression

Cells were seeded in 24-well plates and were transfected at 50–80% confluence with a NKX6-1 expression vector or with empty vector controls using the liposome-mediated transfection method (Invitrogen). To establish cells with stable expression of NKX6-1, MES-SA cells were transfected with the plasmid of choice (pcDNA3.1/NKX6-1 tag V5) for 2 days and then trypsinized and plated at low density. Stable clones were selected by maintaining cells in medium containing G418 antibiotic (Merck).

### RNA interference

Short hairpin RNAs (shRNAs) against human NKX6-1 (TRCN0000017548 and TRCN0000072223) were obtained from the National RNAi Core Facility located at the Institute of Molecular Biology/Genomic Research Center, Academia Sinica, Taiwan. The target sites for the NKX6-1 shRNAs were: 5′‐ CCG GGA AGA CTT TCG AAC AAA CAA ACT CGA GTT TGT TTG TTC GAA AGT CTT CTT TTT‐3′ (TRCN0000017548) and 5′‐ CCG GCC GCT GTA CCC TGC CGC GTA TCT CGA GAT ACG CGG CAG GGT ACA GCG GTT TTT‐3′ (TRCN0000017551). A scrambled shRNA (LacZ) 5′‐TGT TCG CAT TAT CCG AAC CAT‐3′ (TRCN0000072223) was used as a negative control. MES-SA/DX5 cells endogenously expressing NKX6-1 were transfected with different shRNA constructs to evaluate the effects on tumor cells. Stable NKX6-1-knockdown clones were generated by transfecting cells with NKX6-1 or control shRNAs and were selected with puromycin (Invitrogen).

### RNA isolation and reverse transcriptase-PCR

Total RNA was extracted from clinical specimens using TRIzol reagent (Invitrogen), and mRNA was isolated from each cell line sample using the Qiagen RNeasy Kit (Qiagen). An additional DNase I digestion step was added to the RNA isolation procedure to remove DNA and was performed according to the manufacturer’s protocol (Qiagen). Five micrograms of total RNA from each sample were subjected to cDNA synthesis using Superscript III reverse transcriptase (Invitrogen) with random hexamer primers (Promega). cDNA was then PCR amplified with primers specific for NKX6-1 and the glyceraldehyde-3-phosphate dehydrogenase (GAPDH) gene using a PCR Master Mix Reagents Kit (RBC Bioscience). After heating at 95 °C for 10 min, PCR was performed in a thermal cycler (Biometra) for 37 cycles, each of which consisted of denaturation at 95 °C for 30 s, annealing at 58 °C for 30 s, and extension at 72 °C for 30 s, followed by a final 10 min extension at 72 °C. The PCR products were analyzed by electrophoresis on 2% agarose gels (Bioshop).

### Real-time quantitative PCR

Quantitative reverse transcriptase-PCR analysis was performed on a Roche LC480 real-time system. GAPDH was used as an internal control. PCR was performed using a SYBR Green PCR Master Mix Reagent Kit (PE Applied Biosystems). Relative gene expression was determined based on the threshold cycles (Cts) of the genes of interest and the internal control gene. The mRNA levels of the genes of interest are expressed as the ratio of each gene of interest to GAPDH for each sample; mRNA expression was compared between sarcoma cell lines with different constructs. The average Ct value of the GAPDH gene was subtracted from the average Ct value of the gene of interest for each sample. The fold change (2^−△Ct^) in expression of the target gene was calculated relative to the internal control gene (GAPDH) for each sample. The primers used in this study are shown in Additional file 1: Table S1.

### Western blot analysis

Cell pellets were lysed in 10 mM HEPES, 10 mM NaCl, 0.1 mM EDTA, 0.1 mM EGTA, 1% NP-40, 0.5 mM phenylmethylsulfonyl fluoride (PMSF), 0.1 mM DTT, 0.1 mM Na_3_VO_4_, and protease inhibitors. Protein samples (80 μg of each) were separated by SDS-PAGE (10%) and transferred to polyvinylidene difluoride (PVDF) membranes (Millipore). Anti-β-actin antibody was purchased from Abcam (Cambridge, MA), and anti-NKX6-1 antibody was purchased from Cell Signaling (Danvers, MA).

### Cell viability assay

Cells were plated at 2000 cells per well in a 96-well plate for 1 day and then treated with chemotherapeutic drugs and the SHH inhibitor RU-SKI 43 (Cayman) for 96 h. Cell viability was measured using an MTS assay kit (CellTiter 96 AQueous Non-Radioactive Cell Proliferation Assay, Promega). Briefly, 20 μl/well MTS reagent was added to 80 μl of medium containing cells in each well of a 96-well plate and left for 1 h in a humidified incubator at 37 °C and 5% CO_2_. For colorimetric analysis, the absorbance at 490 nm was recorded using a microplate reader (Multiskan EX, Thermo). Each condition was repeated at least 4 times. All cells were harvested at the designated times after treatment.

### Anchorage-independent growth (AIG)

A 2.5 ml base layer of agar (0.7% agar in culture medium) was allowed to solidify in a six-well flat-bottomed plate before the addition of 2 ml of cell suspensions containing 10,000 cells in 0.5% agar. Colonies were allowed to grow for 14–21 days at 37 °C with 5% CO_2_ before imaging. Medium was changed 2 times per week. Plates were stained with p-iodonitrotetrazolium violet (INT, Sigma-Aldrich). Colony numbers in the entire dish were counted.

### In vivo tumor xenograft model

Four week-old athymic nude mice (BALB/cByJNarl) were purchased from the National Laboratory Animal Center (Taipei, Taiwan). Mice were allowed to acclimate to animal housing for 7 days before study. The protocol for this animal experiment was approved by the Institutional Animal Care and Use Committee of the National Defense Medical Center, Taipei, Taiwan. All animal procedures and animal care were performed according to institutional animal research guidelines. A total of 1 × 10^6^ cells were resuspended in 100 μl PBS and subcutaneously (s.c.) injected into each murine flank using a 1-cc syringe with a 29-gauge needle. N = 3 for each transfectant. At the end of the experiment, all mice were euthanized and tumors were harvested.

### Immunohistochemistry

LMS tissues were fixed in 4% paraformaldehyde for 24 h and then placed in 30% sucrose PBS buffer before being embedded at the optimal cutting temperature and frozen. Approximately 5-μm-thick sections were prepared for hematoxylin and eosin (H&E) staining and immunohistochemical analyses. For morphometric lesion analysis, the sections were stained with Mayer’s H&E (Atom Scientific, Manchester, UK). Sections for GLI1 staining were fixed by immersion in ice-cold acetone/methanol (1:1) for 3 min. These sections were incubated in a blocking solution of 3% goat serum in PBS for 1 h at room temperature and then with monoclonal mouse anti-human GLI1 antibody (sc-515751, Santa Cruz) overnight. The sections were then incubated with a horseradish peroxidase-labeled secondary antibody for 1 h at room temperature, and peroxidase activity was visualized using a chromogenic solution of diaminobenzidine at room temperature.

### Statistical analysis

A two-tailed Mann–Whitney *U* test was used to compare data groups for in vitro studies and relative mRNA expression in the different stable transfectants. Standard deviations were used for error bars and various comparisons. Kaplan–Meier analysis, Cox regression and log-rank tests were used to calculate survival and to evaluate differences between overall survival (OS). *p*-values < 0.05 were considered to be statistically significant. RNA expression profiles (RNA-sequencing data) of LMS cases were obtained from The Cancer Genome Atlas (TCGA). LMS classification was defined by TCGA. In the survival analysis, patients with FPKM > 66th percentile (1/3) were defined as having high expression, while patients with FPKM ≤ 66th percentile (2/3) were defined as having low expression. The results and clinical data were downloaded from the Broad Institute GDAC Firehose (http://gdac.broadinstitute.org/) and used in compliance with TCGA’s data usage policy.

## Results

### Clinical correlation of the NKX transcription factor family in LMS

**Fig. 1:**
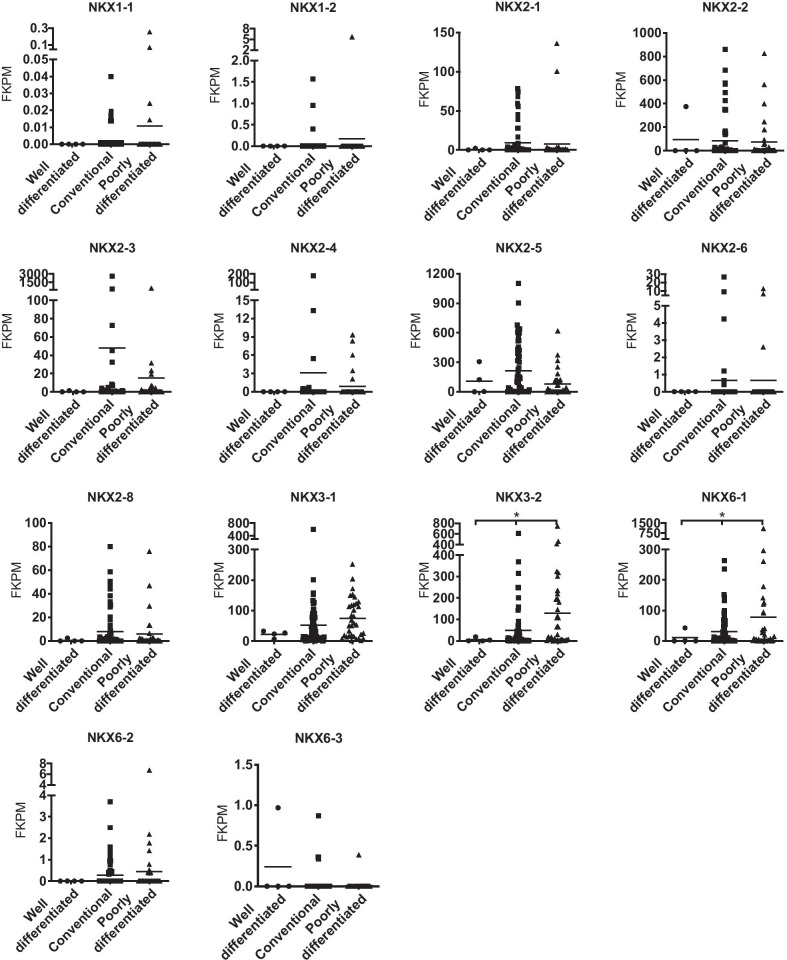
RNA expression of *NKX* family members in LMS. The expression levels of 14 NKX family mRNAs in well-differentiated, conventional, and poorly differentiated LMS from TCGA. *p < 0.05

**Fig. 2:**
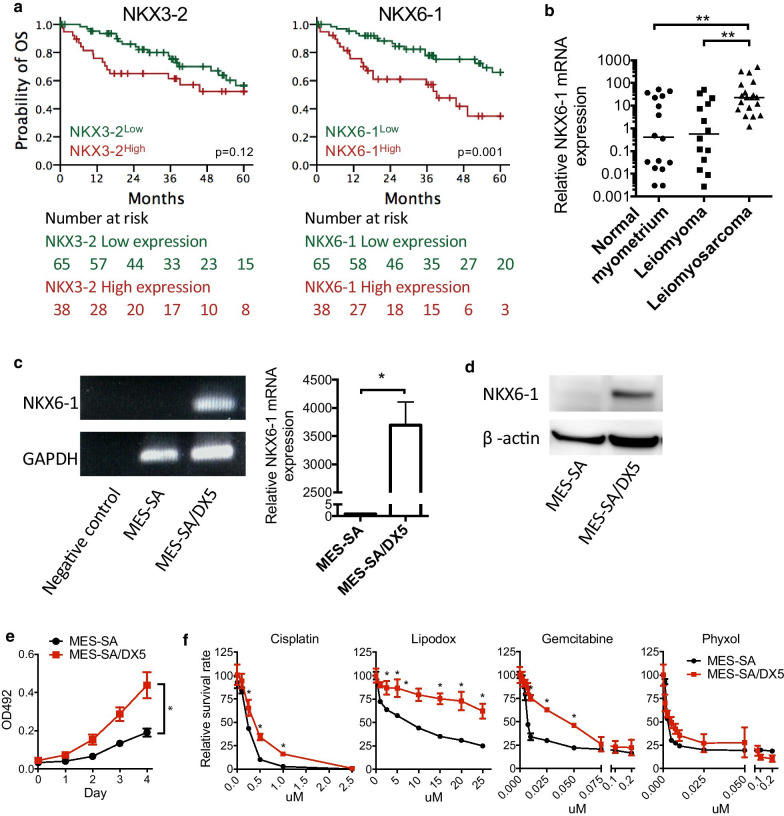
*NKX6-1* expression is upregulated in LMS tissues and correlates with malignancy. **a** Kaplan–Meier analysis of *NKX3-2* and *NKX6-1* in LMS patients from a dataset in The Cancer Genome Atlas (TCGA). **b** NKX6-1 mRNA levels in 49 human uterine tissues, including normal myometrium (n = 16), leiomyoma (n = 14), and LMS (n = 19). *NKX6-1* mRNA **c** and protein **d** expression was determined by qRT-PCR and immunoblotting in MES-SA and MES-SA/DX5 cells. The NKX6-1/β-actin protein expression ratios were 0.31 and 1.19, respectively. In vitro growth curves **e** and analyses of resistance to cisplatin, lipodox, gemcitabine, and phyxol **f** in the MES-SA parental and MES-SA/DX5 daughter cell lines. *p < 0.05, **p < 0.01

To explore the role of the NKX family in LMS, we analyzed the expression levels of *NKX* family mRNAs in well-differentiated, conventional, and poorly differentiated LMS cases from TCGA. Among the 14 *NKX* genes, the mRNA expression levels of *NKX3-2* and *NXK6-1* were higher in poorly differentiated LMS than in well-differentiated or conventional LMS (Fig. 1). LMS patients with both high and low *NKX3-2* mRNA expression levels had similar median 5 year OS durations (*NKX3-2*^high^ vs. *NKX3-2*^low^, > 60 months vs. > 60 months, hazard ratio (HR) = 1.7, 95% confidence interval (CI): 0.9–3.3) and 5 year survival rates (*NKX3-2*^high^ vs. *NKX3-2*^low^, 53 vs. 57%). However, compared to *NKX6-1*^low^ patients, LMS patients with high *NKX6-1* mRNA expression had a worse prognosis, with a median 5 year OS of 39.2 months (*NKX6-1*^high^ vs. *NKX6-1*^low^, 39.2 vs. > 60 months, HR = 2.9, 95%: 1.5–5.7) and a 5 year survival rate of 34% (*NKX6-1*^high^ vs. *NKX6-1*^low^, 34% vs. 66%). (Fig. 2a). We further found that *NKX6-1* expression was higher in LMS (n = 19) than in nonmalignant leiomyomas (n = 14) or normal myometrium (n = 16) (Fig. 2b).

### NKX6-1 promotes malignant phenotypes in LMS cell lines

To determine the functional effects of NKX6-1 in LMS, we chose the human sarcoma cell line MES-SA and its chemoresistant daughter cell line MES-SA/DX5 [23]. We observed that NKX6-1 mRNA (Fig. 2c) and protein (Fig. 2d) expression levels were higher in MES-SA/DX5 cells than in MES-SA cells. Interestingly, malignant phenotypes, including proliferation and resistance to cisplatin, lipodox, and gemcitabine, were significantly higher in MES-SA/DX5 cells than in MES-SA cells (Fig. 2e, f).

**Fig. 3:**
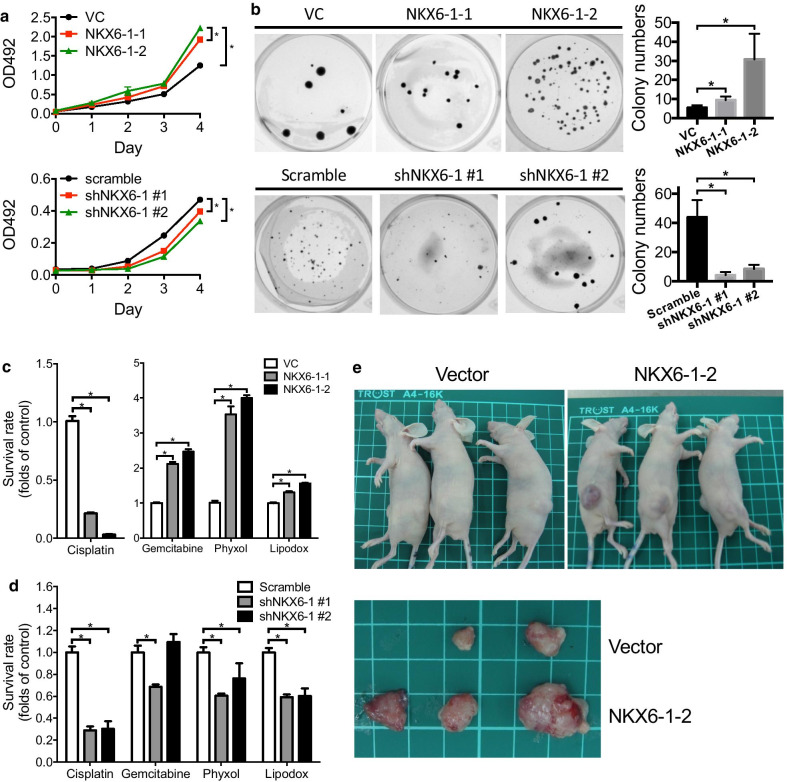
NKX6-1 promotes malignancy in LMS cells. Overexpression of *NKX6-1* in MES-SA LMS cells increases proliferation and colony formation. Knockdown of *NKX6-1* in MES-SA/DX5 cells, by contrast, decreases proliferation. **a** A cell proliferation assay was performed using the MTS assay. **b** Colony formation ability was assessed using the AIG assay. *NKX6-1*-overexpressing MES-SA cells **c** and NKX6-1 knockdown MES-SA/DX5 cells **d** were evaluated for resistance to cisplatin, gemcitabine, phyxol, and doxorubicin. The concentrations of cisplatin, gemcitabine, phyxol, and doxorubicin in **c** are 0.33, 0.01, 0.005 and 7.5 μM, respectively (the IC_50_ in MES-SA-VC cells). The concentrations of cisplatin, gemcitabine, phyxol, and doxorubicin in **d** are 0.5, 0.05, 0.0125 and 30 μM, respectively (the IC_50_ in MES-SA/DX5-Scramble cells). **e** In vivo tumor growth of *NKX6-1*-overexpressing MES-SA cells. *p < 0.05

Thereafter, we generated NKX6-1 gain-of-function cells by transfecting full-length NKX6-1 into the MES-SA cell line and loss-of-function cells by transfecting NKX6-1 shRNAs into the MES-SA/DX5 cell line (confirmed by western blotting in Additional file 2: Figure S1). Overexpression of NKX6-1 protein enhanced numerous malignant phenotypes, including proliferation, colony formation, and resistance to gemcitabine, phyxol, and lipodox (Fig. 3a–c). Conversely, knockdown of NKX6-1 in MES-SA/DX5 cells suppressed proliferation, colony formation, and resistance to cisplatin, gemcitabine, phyxol, and lipodox (Fig. 3a, b, d). Tumor xenograft experiments demonstrated that overexpression of NKX6-1 enhanced tumor growth in vivo (Fig. 3e). These results demonstrate that NKX6-1 plays an oncogenic role in LMS.

### NKX6-1 enhances stem cell properties and regulates the SHH and NOTCH pathways in LMS cells

**Fig. 4:**
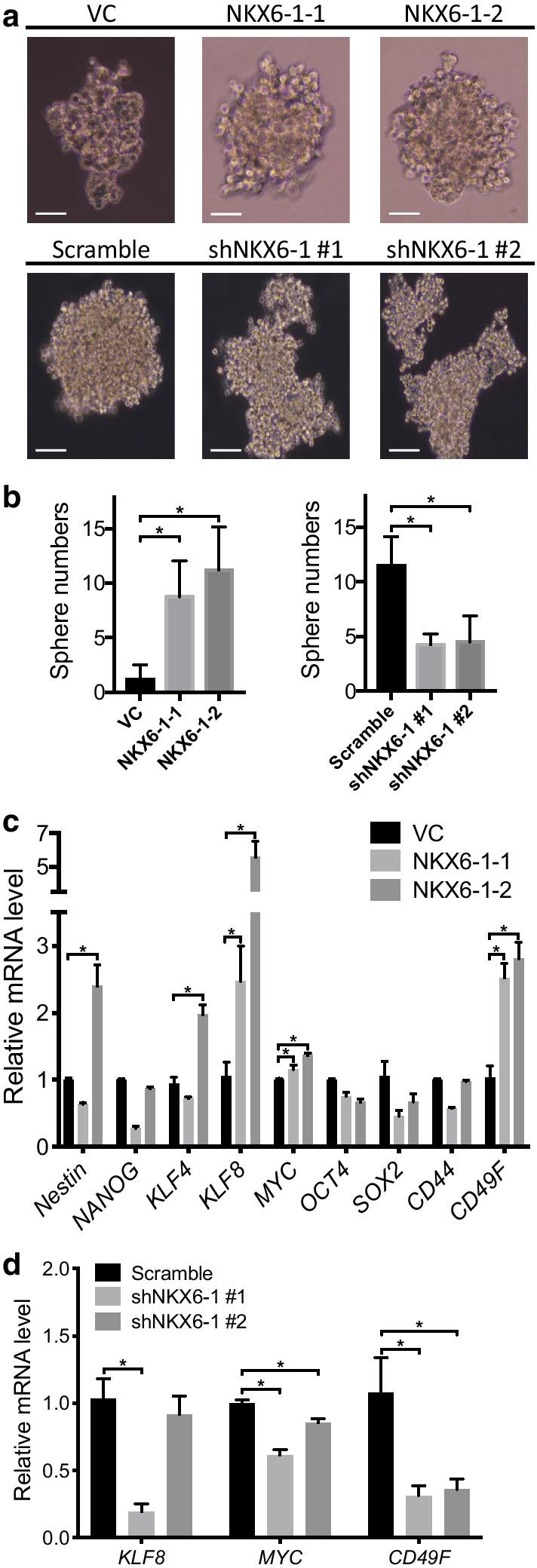
NKX6-1 promotes cancer stemness properties in LMS cells. Cells were seeded in ultralow attachment plates to assess sphere-forming ability. **a** Representative spheroid images from different NKX6-1 transfectants. NKX6-1-overexpressing MES-SA cells formed spheroids with round morphology (upper), and NKX6-1 knockdown MES-SA/DX5 cells formed clusters of loosely associated cells (lower). The scale bar represents 100 μm. **b** The number of spheres was quantified using ImageJ software. The total number of spheres increased upon NKX6-1 overexpression in MES-SA cells and decreased upon NKX6-1 knockdown in MES-SA/DX5 cells. The expression of cancer stemness-related genes correlated with sphere formation ability. **c** qRT-PCR was performed to evaluate stemness markers (including NESTIN, NANOG, KLF4, KLF8, MYC, OCT, SOX2, CD44, and CD49F) in MES-SA cells transfected with either NKX6-1 or vector control. **d** Expression of KLF8, MYC, and CD49F in MES-SA/DX5 cells transfected with either shNKX6-1 or scrambled control was determined by qRT-PCR. *p < 0.05

Since NKX6-1 was reported to be related to stem cell differentiation [24, 25], we explored the role of NKX6-1 in cancer stemness. Our results demonstrated that overexpression of NKX6-1 in MES-SA cells enhanced sphere formation while knockdown of NKX6-1 in MES-SA/DX5 cells suppressed sphere formation (Fig. 4a, b). Next, we assessed expression of stemness markers, including *NESTIN*, *NANOG*, *KLF4*, *KLF8, MYC*, *OCT4*, *SOX2*, *CD44*, and *CD49F*, following the manipulation of *NKX6-1* expression in MES-SA and MES-SA/DX5 cells. These results showed that expression of the stemness markers *KLF8*, *MYC*, and *CD49F* was increased in *NKX6-1*-overexpressing MES-SA cells and reduced after knockdown of NKX6-1 in MES-SA/DX5 cells (Fig. 4c, d). These results suggest that NKX6-1 plays a phenotype-regulatory role in LMS.

**Fig. 5:**
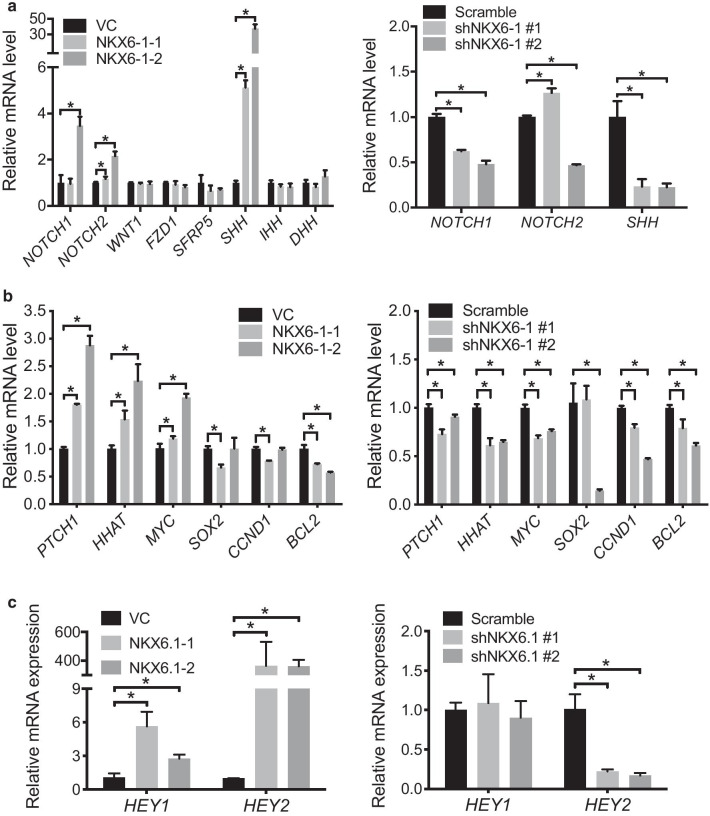
*NKX6-1* mediates the activation of stemness, SHH, and Notch signaling pathways. qRT-PCR analysis of Notch, WNT and SHH signaling. **a** qRT-PCR for *NOTCH1*, *NOTCH2*, *WNT1*, *FZD1*, *SFRP5*, *SHH*, *IHH*, and *DHH* in NKX6-1-overexpressing MES-SA cells. **b** qRT-PCR for *NOTCH1*, *NOTCH2*, and *SHH* in NKX6-1 knockdown MES-SA/DX5 cells. **c** Expression of SHH **c** and NOTCH **d** downstream genes was determined by qRT-PCR. *p < 0.05

To elucidate the regulatory effects of NKX6-1 on stemness, potential relevant signaling pathways, such as WNT, Hedgehog and Notch, were evaluated [26]. These results demonstrated that the SHH and NOTCH1/2 signaling pathways were upregulated in NKX6-1-overexpressing MES-SA cells and downregulated in NKX6-1-knockdown MES-SA/DX5 cells (Fig. 5a). Genes downstream of SHH signaling, such as PTCH1 and MYC, and SHH-related acyltransferases, such as HHAT, were upregulated upon NKX6-1 overexpression in MES-SA cells. Conversely, PTCH1, HHAT, MYC, SOX2, CCND1 and BCL2 expression levels were decreased after NKX6-1 knockdown in MES-SA/DX5 cells (Fig. 5b). SOX2, CCND1, and BCL2 demonstrated the same trend in opposing treatment conditions, which may be due to crosstalk between signaling pathways. These regulatory pathways, such as PI3K/AKT and SOX2 [27], DNA damage/ATM and CCND1[28], JAK2/ERK and BCL2 [29], may compensate for the effects of NKX6-1, though these compensatory effects should be investigated in future studies. Moreover, the NOTCH signaling downstream genes *HEY1* and *HEY2* were upregulated in NKX6-1-expressing MES-SA cells, while *HEY2* was downregulated in NKX6-1-knockdown MES-SA/DX5 cells (Fig. 5c). Taken together, these data indicate that NKX6-1 promotes stemness phenotypes by regulating the SHH and NOTCH signaling pathways in LMS cells.

### NKX6-1 upregulates SHH signaling and sensitizes LMS cells to an SHH inhibitor

**Fig. 6:**
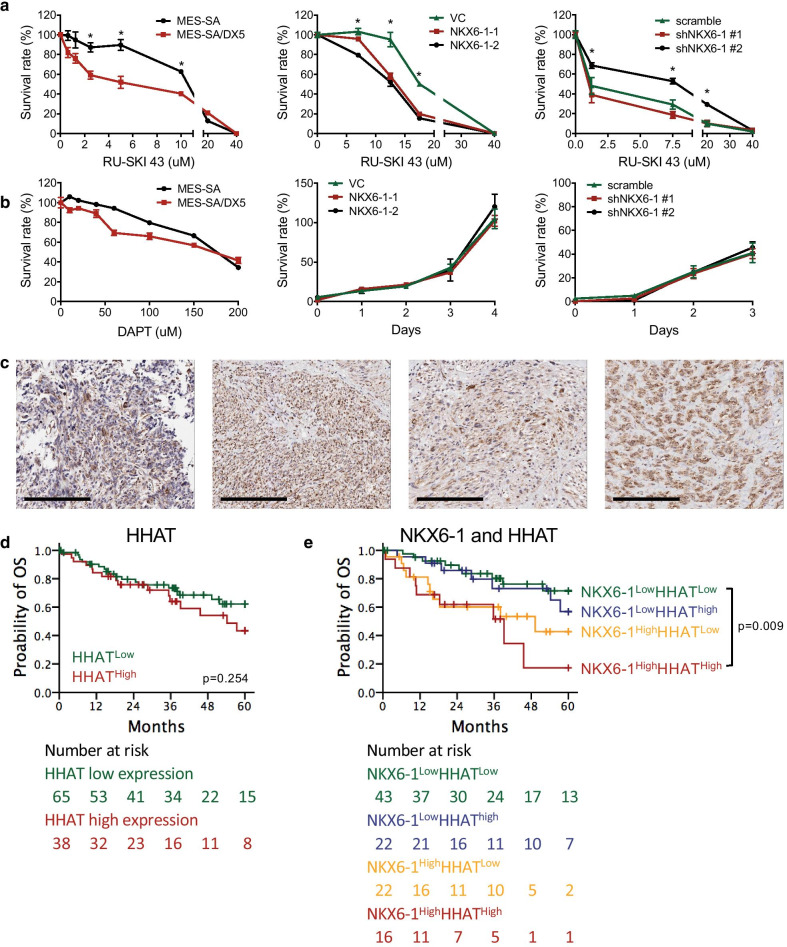
NKX6-1 activation enhances chemosensitivity to an SHH inhibitor. Dose–response curves (**a** and **b**, left panel) and growth curves (**b**, middle and right panel) of LMS cells and NKX6-1-overexpressing vs. -knockdown cells treated for 96 h with SHH (RU-SKI 43) and NOTCH (DAPT) inhibitors. **c** Immunohistochemical analysis of GLI1 protein expression in LMS tissue from four patients. The scale bar represents 200 μm. **d** Kaplan–Meier analysis of overall survival stratified by HHAT expression in LMS patients from the TCGA dataset. **e** Kaplan–Meier analysis of overall survival stratified by combined NKX6-1 and HHAT expression in LMS patients from the TCGA dataset. *p < 0.05

To determine whether the SHH and/or NOTCH pathways could be potential therapeutic targets in NKX6-1-overexpressing LMS cells, we tested the effects of the SHH inhibitor RU-SKI 43 (an HHAT inhibitor) [30] and the NOTCH inhibitor DAPT [31]. We found that LMS cells with higher *NKX6-1* expression were more sensitive to RU-SKI 43. MES-SA/DX5 cells were more sensitive than MES-SA cells. *NKX6-1*-overexpressing MES-SA cells were more sensitive than control cells. *NKX6-1* knockdown MES-SA/DX5 clone 1 was more sensitive than control cells, and there was no sensitivity difference between clone 2 and control cells (Fig. 6a). *MYC* and *SOX2* were downregulated in both cell lines after treatment (Additional file 2: Figure S2). Moreover, other gynecological cancer cell lines with higher NKX6-1 expression were more sensitive to RU-SKI 43 (Additional file 2: Figure S3). Treatment with DAPT inhibited cell proliferation in vitro; however, it had no significant effects on the various NKX6-1 transfectants (Fig. 6b), suggesting that NKX6-1 is not involved in the molecular mechanisms targeted by DAPT. Recent studies have shown that the SHH signaling pathway is implicated not only in cancer cells but also in stromal cells [32]. We evaluated GLI1 protein expression in LMS tissue by immunohistochemistry and demonstrated that the expression of GLI1 protein in LMS cancer cells (Fig. 6c and Additional file 2: Figure S4) is consistent with that of a previous study indicating the expression of GLI in LMS tissue [33, 34]. Moreover, the SHH downstream genes BCL-2, c-MYC and CCND1 were expressed in LMS tissues (Additional file 2: Figure S5). All these data suggested that SHH signaling is activated in LMS cells.

Since RU-SKI 43 suppresses the SHH acyltransferase *HHAT*, we assessed *HHAT* clinical relevance and prognostic markers in LMS patient data from the TCGA database [35]. We found that the expression of *HHAT* alone was not associated with OS (Fig. 6d). LMS patients with high HHAT mRNA expression had a median 5 year OS of 54.2 vs. > 60 months (HR = 1.5, 95% Cl: 0.8–2.9), and a 5 year survival rate of 45 vs. 61% compared to *HHAT*^low^ LMS patients. Interestingly, combined assessment of *HHAT* and *NKX6-1* demonstrated that patients with the highest expression of both *NKX6-1* and *HHAT* (i.e., *NKX6-1*^high^*HHAT*^high^) had the worst survival outcomes (Fig. 6e), with a median 5 year OS of 39.2 vs. > 60 months (HR = 4.1, 95% CI: 1.6–10.4) and a 5 year survival rate of 26 vs. 71.0% compared to *NKX6-1*^low^*HHAT*^low^ LMS patients.

## Discussion

**Fig. 7:**
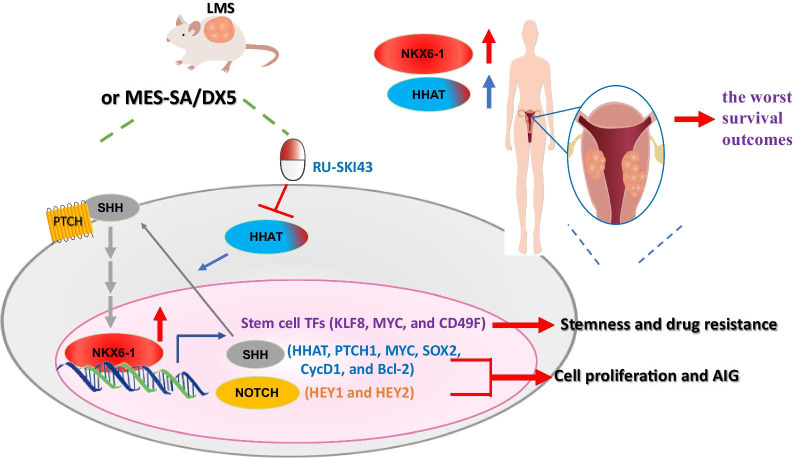
Proposed model of NKX6-1-mediated stemness signaling and cancer stemness properties in LMS. NKX6-1 activates SHH and NOTCH but not WNT, resulting in enhanced malignant phenotypes and poor prognosis in LMS. Inhibition of SHH but not NOTCH inhibits cell growth, suggesting the potential of SHH inhibitors for the treatment of LMS

LMS originates from undifferentiated mesenchymal cells and exhibits highly aggressive behavior, characterized by high rates of recurrence and metastasis. Neither chemotherapy nor radiotherapy has been shown to improve overall survival, thus posing a major challenge to adjuvant treatment in LMS patients. Whether NKX6-1 is important in the pathogenesis of LMS and regulates SHH signaling has not been reported. In the present study, we investigated how oncogenic *NKX6-1* confers poor prognosis in LMS and how NKX6-1 regulates cancer stem cells through activation of the SHH and NOTCH pathways. Our findings reveal novel molecular insights into LMS and lay a foundation for future diagnostic and therapeutic improvements (Fig. 7).

The relationship between SHH and *NKX6-1* is interesting. The SHH pathway plays a crucial role in embryonic vertebral development and tissue homeostasis [36]. In addition, *NKX6-1* expression controls fate specification and differentiation in cells surrounding SHH-expressing cells. While a SHH-independent pathway appears to regulate *NKX6-1* expression in the foregut [37], SHH signaling is required for *NKX6-1* expression in the ventral neural tube and spinal meninges. Previous reports have demonstrated that the *NKX* gene family is expressed in the medial neural plate above the *SHH*-expressing axial mesendoderm [38, 39]. Similar to many embryonic signaling pathways in cancer, the SHH signaling pathway plays important roles in promoting oncogenesis and tumor growth and progression. The dysregulated activation of SHH signaling is implicated in several cancers and has been linked to the maintenance of cancer stem-like cells, which are associated with the development of therapeutic resistance [40–45]. Interestingly, our study found that overexpression of *NKX6-1* modulates SHH pathway genes and promotes cancer stemness, indicating a novel reciprocal regulatory axis between *NKX6-1* and SHH signaling in cancer.

Previous developmental biology studies have shown that Nkx transcription factors act with coactivators or corepressors to regulate tissue-specific gene expression and development. NKX2-5 interacts with HAND2 and GATA4 to promote gene expression, cardiomyocyte differentiation and chamber identity [46]. Nkx2.2 interacts with Grg3 and functions as a transcriptional repressor during islet beta-cell specification in the pancreas [47]. Nkx2.1 interacts with Gata6 to direct pulmonary epithelial differentiation and development [48]. The partners of NKX6-1 should be evaluated in the future.

The different roles of *NKX6-1* suggest a bifunctional divergence whereby downstream target genes can be activated or repressed in a cell type-dependent manner [49]. In cervical cancer, *NKX6-1* plays a tumor suppressive role by suppressing the epithelial-to-mesenchymal transition and cancer metastasis [50]. However, in other contexts *NKX6-1* behaves as an oncogene [51–53]. *NKX6-1* upregulates mesenchymal markers, facilitates disease progression and is associated with poor prognosis in patients with primary hepatocellular carcinoma [51]. In breast cancer, *NKX6-1* increases IL-6 expression and promotes cell proliferation through an *NKX6-1*/IL-6 network [52]. Moreover, immunohistochemical staining for *NKX6-1* could be an effective marker for pancreatic and duodenal neuroendocrine tumors [53]. *NKX6-1* methylation status is an indicator of survival outcome and could inform treatment selection in stage III colon cancer [19]. Our current study indicates that *NKX6-1* plays an oncogenic role in LMS.

Through molecular characterization and analyses of LMS patient prognosis in public datasets, we provide evidence to support the oncogenic role of *NKX6-1*, which serves as a novel biomarker to predict prognosis and guide precision medicine against LMS. A prognostic marker can help clinicians and patients estimate OS and progression-free survival, helping to inform therapeutic decisions. However, few models have been proposed to predict the prognosis of LMS. Cohen et al. observed the association between tumor infiltrating CD8 cytotoxic lymphocytes, PD-L1 staining, expression of mismatch repair-related proteins (MSH2, MLH1, MSH6 and PSM2) and survival in patients with LMS [54]. Studies from Xue et al. suggested that age greater than 60 years, high tumor grade, distant metastasis, tumor size ≥ 5 cm, and lack of surgery were associated with decreased OS and cancer-specific survival [55]. These models were characterized by complexity and clinical impracticality. The results obtained in our study from both cell lines and patient samples provide strong evidence to support the oncogenic role of *NKX6-1* in LMS and suggest that NKX6-1 could serve as a novel biomarker and guide the treatment of LMS.

The SHH signaling pathway has been implicated in the progression of cancers. In an essential step for SHH pathway activation, *HHAT* catalyzes the transfer of the fatty acid palmitate onto SHH-related signaling proteins [56]. Therefore, the potential of HHAT inhibition has been tested in various cancers, including breast cancer [57] and pancreatic ductal adenocarcinoma [58]. Recently, SHH signaling activation was shown to induce undifferentiated soft tissue sarcomas in a mouse model. [59]. Moreover, high expression of SHH signaling pathway proteins has been observed in LMS patients [33]. Based on the present and previous studies, it is important to consider a new therapeutic intervention that targets the SHH pathway to block the progression of this type of malignancy [60]. Here, we have identified a relationship between *NKX6-1* expression and SHH signaling activation in cancer cells and we therefore propose that SHH inhibition may represent a feasible strategy for the treatment of *NKX6-1*^high^ LMS patients.

Primary cilia play important roles in the activation of the Hedgehog pathway. Upon sensing extracellular signals, the components of the hedgehog pathway, including GLI transcription factors, accumulate at primary cilia [61]. In rhabdomyosarcoma and Ewing sarcoma, GLI1 is upregulated and contributes to drug resistance [62]. Our results also demonstrated increased GLI1 expression in LMS tissue. However, cilia may play tumor suppressive or oncogenic roles depending on SHH pathway regulation [63]. Various types of neoplasms, including ovarian, pancreatic, and renal cancers, fail to express cilia and centrioles [64], indicating that the role of cilia dysfunction in the tumorigenic process is complex and deserves further investigation in the future.

Some limitations should be mentioned in our study. First, the role of *NKX6-1*, *SHH*, and *NOTCH* in the in vivo chemoresistance of LMS were not evaluated in the current study. Moreover, the inconsistent effects of *NKX6-1* overexpression and knockdown during cisplatin treatment in human sarcoma cells require further investigation. The current study did not use omics-level approaches to analyze the putative genes regulated by NKX6-1; this should be evaluated in a future study. Additionally, manipulation of *NKX6-1* exhibits inconsistent effects on the expression of NOTCH1 and NOTCH2. Although inhibition of NOTCH signaling has modest effects on LMS prognosis with either partial response or stable disease in approximately 40% of patients [65, 66], our unpublished results demonstrate that NOTCH inhibition displays *NKX6-1*-independent chemosensitizing effects in LMS. Further studies should be conducted to uncover the mechanisms by which NOTCH signaling promotes stemness and to determine how we can best incorporate them into LMS therapeutics. Even with these limitations, our studies reveal novel roles of *NKX6-1* and *SHH* signaling in the pathogenesis of LMS, which opens new avenues for the treatment of LMS.

## Conclusions

Although large-scale studies are needed, our study demonstrated that NKX6-1 expression regulates the cancer stemness phenotype through activation of the SHH pathway and is correlated with poor prognosis in LMS. Along with the diagnostic potential of detecting NKX6-1 in cancer tissues, our findings suggest that SHH inhibitors could be applied to treat LMS.

## Supplementary Information

**Additional file 1: Table S1.** Primers used in the present study.

**Additional file 2: Figure S1. **NKX6-1 protein expression in the different stable transfectants.** Figure S2. **Effects of RU-SKI 43 treatment on SHH downstream genes. **Figure S3. **Increased NKX6-1 expression correlates with increased RU-SKI 43 resistance in LMS cell lines.** Figure S4. **GLI1 protein expression in LMS. **Figure S5. **IHC analysis of BCL-2, c-MYC and Cyclin D1 expression in LMS.

## Data Availability

All data generated or analyzed during this study are included in this published article.

## Abbreviations

BCL2: B-cell lymphoma 2
CCND1: Cyclin D1
DHH: Desert hedgehog
FZD1: Frizzled-1
HHAT: Hedgehog Acyltransferase
IHH: Indian hedgehog
MYC: MYC Proto-Oncogene, BHLH Transcription Factor
NKX6-1: NK6 Homeobox 1
NOTCH1: Notch Receptor 1
NOTCH2: Notch Receptor 2
PTCH1: Patched 1
SFRP5: Secreted Frizzled Related Protein 5
SHH: Sonic hedgehog
SOX2: SRY-Box Transcription Factor 2
