# Supplementary material for: NKX6-1 mediates cancer stem-like properties and regulates sonic hedgehog signaling in leiomyosarcoma
Source: J Biomed Sci. 2021 Apr 27;28:32. doi: 10.1186/s12929-021-00726-6 (PMC8077933; doi:10.1186/s12929-021-00726-6)
Supplement: Supplementary file 1 — Additional file 1: Table S1. Primers used in the present study. [file 12929_2021_726_MOESM1_ESM.pdf]

Gene	Forward sequence	Reverse sequence
BCL2	GGTGGGGTCATGTGTGTGG	CGGTTCAGGTACTCAGTCATCC
CCND1	CAATGACCCCGCACGATTTC	CATGGAGGGCGGATTGGAA
CD44	AAGGTGGAGCAAACACAACC	ACTGCAATGCAAACTGCAAG
CD49F	CAGTGGAGCCGTGGTTTTG	CCACCGCCACATCATAGCC
FRIZZLED1	GAGCCCATACTCATCAAGTACCG	CCTCGGGAGAGATGCACAC
GAPDH	ACCCACTCCTCCACCTTTGACG	TCTCTTCCTCTTGTGCTCTTG
HHAT	GTCTGGCCCGATACTTCTCC	GGATCAGCATCGAGGTGGAAC
IHH	AACTCGCTGGCTATCTCGGT	GCCCTCATAATGCAGGGACT
KLF4	GGGAGAAGACACTGCGTCA	GGAAGCACTGGGGGAAGT
KLF8	GTCAGTCTGCCAAATAAGATGGG	ATGGAGGTGGGGTCAACTTTC
MYC	TCCCTCCACTCGGAAGGAC	CTGGTGCATTTTCGGTTGTTG
NANOG	ATTCAGGACAGCCCTGATTCTTC	TTTTTGCGACACTCTTCTCTGC
NESTIN	TGAGAGGCCTGGCCCGCAA	GGCACAAAAGCCAGCATGTC
NKX6.1	CGTTGGGGATGACAGAGAGT	CGAGTCCTGCTTCTTCTTGG
OCT4	GAAACCCACACTGCAGATCA	CGGTTACAGAACCACACTCG
PTCH1	ACTTCAAGGGGTACGAGTATGT	TGCGACACTCTGATGAACCAC
SFRP5	CAGATGTGCTCCAGTGACTTTG	AGAAGAAAGGGTAGTAGAGGGAG
SHH	CTCGCTGCTGGTATGCTCG	ATCGCTCGGAGTTTCTGGAGA
SOX2	GGGAAATGGGAGGGGTGCAAAAGAGG	TTGCGTGAGTGTGGATGGGATTGGTG
WNT1	CGGCGTTTATCTTCGCTATCA	GCAGGATTCGATGGAACCTTCT

Supplemental table 1. Primers used in the present study.
