# Supplementary material for: NKX6-1 mediates cancer stem-like properties and regulates sonic hedgehog signaling in leiomyosarcoma
Source: J Biomed Sci. 2021 Apr 27;28:32. doi: 10.1186/s12929-021-00726-6 (PMC8077933; doi:10.1186/s12929-021-00726-6)
Supplement: Supplementary file 2 — Additional file 2: Figure S1. NKX6-1 protein expression in the different stable transfectants. Figure S2. Effects of RU-SKI 43 treatment on SHH downstream genes. Figure S3. Increased NKX6-1 expression correlates with increased RU-SKI 43 resistance in LMS cell lines. Figure S4. GLI1 protein expression in LMS. Figure S5. IHC analysis of BCL-2, c-MYC and Cyclin D1 expression in LMS. [file 12929_2021_726_MOESM2_ESM.pdf]

Supplemental Figure

A

B

C

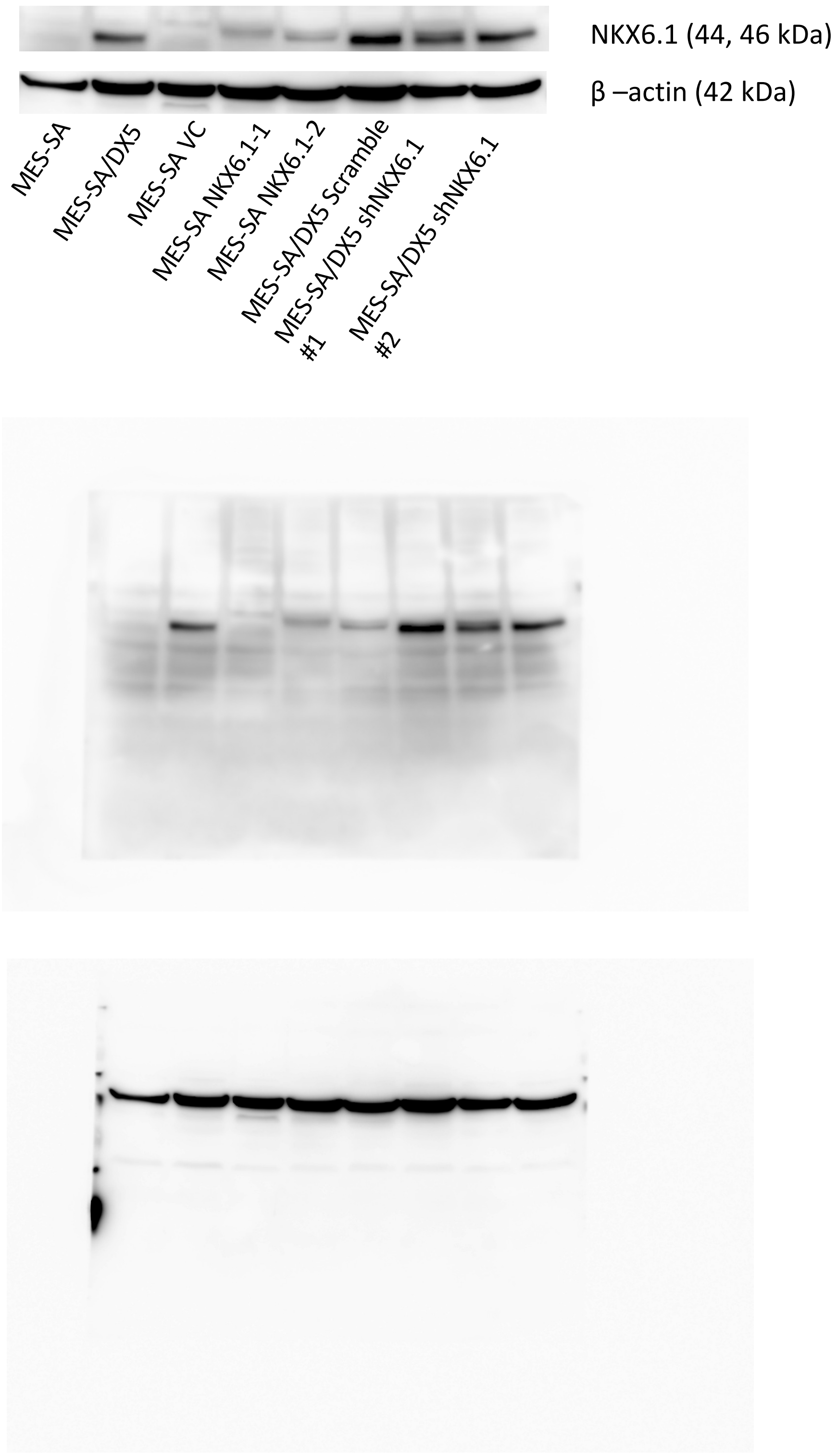

Supplemental Figure 1. NKX6-1 protein expression in the different stable transfectants. (A) Western blot showing NKX6-1 and beta-actin protein expression. Full-length blots for NXK6-1 and beta-actin are shown in (B) and (C), respectively.

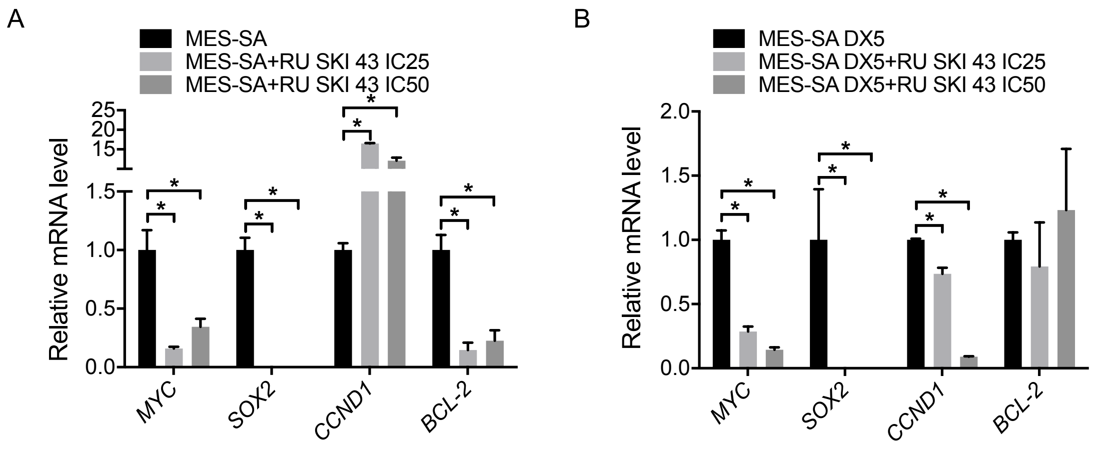

Supplemental Figure 2. Effects of RU-SKI 43 treatment on SHH downstream genes. Expression of the SHH downstream genes *MYC, SOX2, CCND1* and *BCL-2* was evaluated by qRT-PCR following RU-SKI 43 treatment in the different MES-SA (A) and MES-SA/DX5 (B) transfectants.

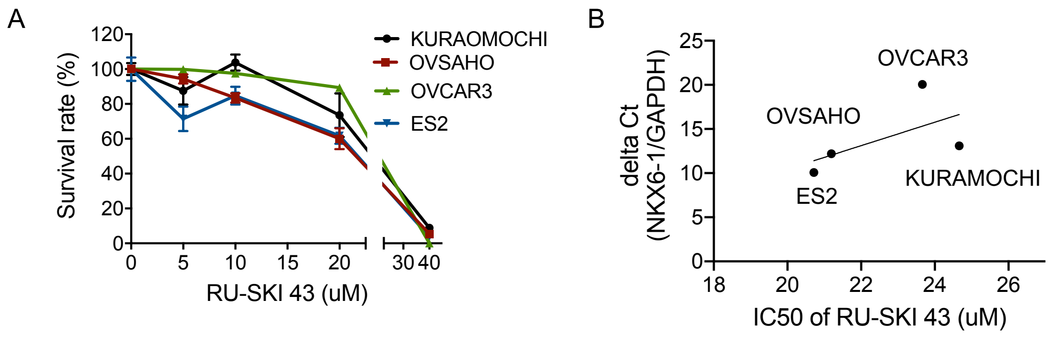

Supplemental Figure 3. Increased NKX6-1 expression correlates with increased RU-SKI 43 resistance in LMS cell lines. (A) Dose-response curves of cell lines treated with RU-SKI 43. (B) Correlation between NKX6-1 expression (Y-axis) and RU-SKI 43 IC_{50} (X-axis) in various cell lines. NKX6-1 expression was calculated using the delta Ct method (NKX6-1 Ct – GPADH Ct). The RU-SKI 43 IC_{50} was calculated from (A).

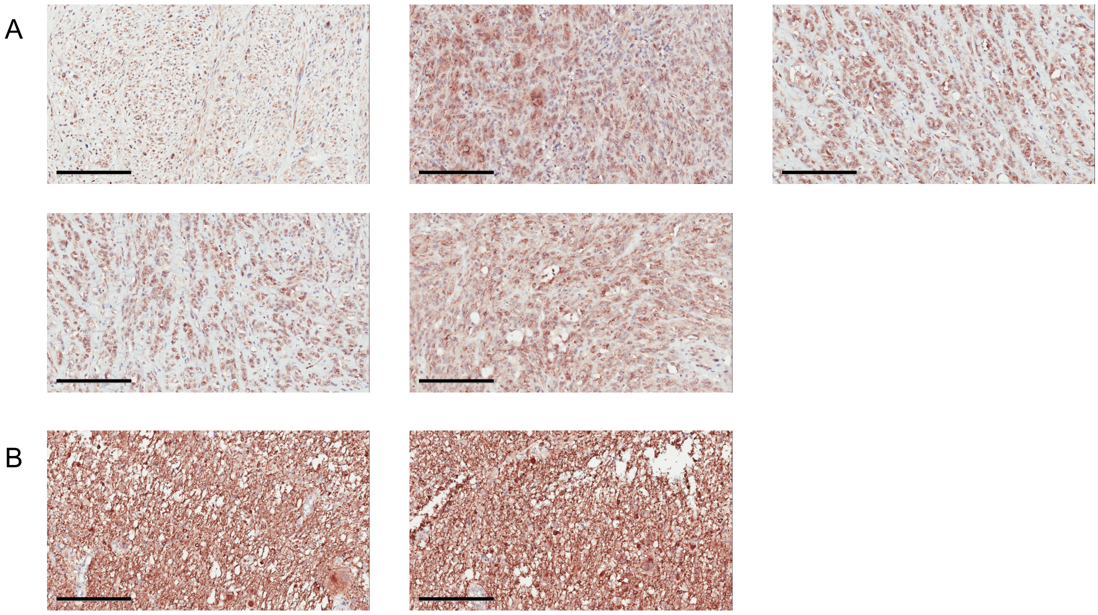

Supplemental Figure 4. GLI1 protein expression in LMS. Representative immunohistochemical analysis of GLI1 protein expression in tumor samples. (A) LMS tissues (n=5). (B) Glioblastoma multiforme tissues (n=2, positive control). The scale bar represents 200 μm.

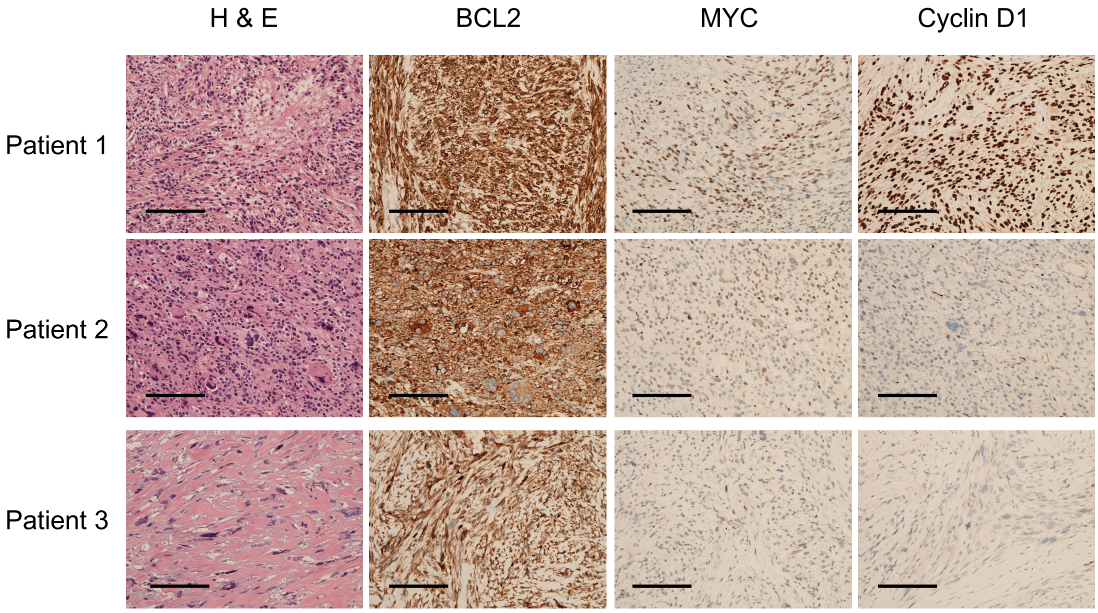

Supplemental Figure 5. IHC analysis of BCL-2, c-MYC and Cyclin D1 expression in LMS. The scale bar represents 200 μm.
